# Comparative Evaluation of Mechanical Properties and Wear Ability of Five CAD/CAM Dental Blocks

**DOI:** 10.3390/ma12142252

**Published:** 2019-07-12

**Authors:** Ruizhi Yin, Yong-Seok Jang, Min-Ho Lee, Tae-Sung Bae

**Affiliations:** Department of Dental Biomaterials, School of Dentistry, Chonbuk National University, 664-14, Duckjin-dong, Jeonju-city 561-756, Korea

**Keywords:** hybrid ceramic, nano-composite resin, Vickers hardness test, wear test, surface roughness, biaxial flexural strength

## Abstract

This study compares the mechanical properties and wear ability of five CAD/CAM (computer-aided design/computer-aided manufacturing) millable dental blocks. All the discs, including Amber Mill Hybrid, Vita Enamic, Katana Avencia, Lava Ultimate, and Amber Mill, were cut in dimensions of 1.2 mm in thickness and 12 mm in diameter, polished to a machined surface, and immersed in distilled water for seven days. Vickers hardness was measured and the indentations were observed using microscope. The discs were brushed under a 150 g load. Mean surface roughness (Ra) and topography were determined after 100,000 cycles. Finally the biaxial flexure strength of the discs was measured and the broken surfaces were observed using scanning electron microscopy (SEM). The data was subjected to Weibull analysis. All data were analyzed by one-way analysis (ANOVA). The results of Vickers hardness are shown as: Amber Mill > Vita Enamic > Amber Mill Hybrid > Lava Ultimate > Katana Avencia. Katana Avencia showed the highest volume percentage reduction and the roughest surface after toothbrushing. The biaxial flexural strength is shown as: Amber Mill > Katana Avencia > Lava Ultimate > Amber Mill Hybrid > Vita Enamic. All the tested materials exhibited varying degrees of mass loss and surface roughness. The properties of the composite materials are related to the filler content, filler volume, and polymerization methods.

## 1. Introduction

The past three decades have witnessed significant advances in both dental restorative materials and dental restorative technology. On the one hand, such advancements are due to rapid developments in chairside computer technology and the combination of computer technology and dental restoration technology, such as in computer-aided design/computer-aided manufacturing (CAD/CAM) image processing and design and 3D printing. On the other hand, the advancements can also be attributed to the development of restorative materials itself.

Currently, there are three main types of dental block materials used under clinical conditions, which can be aesthetically processed by CAD/CAM: Ceramics, glass ceramics, and resin composites [1]. Ceramics require additional processing such as specialized sintering and glazing after grinding [2,3], while glass ceramics and resin composites conveniently do not require these steps and can be quickly fabricated directly with oral clinical-use equipment [4]. In this context, it has been reported in several studies that traditional ceramics such as zirconia have high mechanical strength and excellent wear resistance against contact with teeth [5,6]. However, some studies have posited that the ceramic failure rate is extremely high due to the brittleness of the ceramics and potential wear on opposite dentition [7,8,9,10]. Meanwhile, it is of note that most of the earlier composite resins were composed of resin-based ceramics. However, some studies have reported that their large surface wear, relatively rough surface structure, and low mechanical strength can lead to their failure [11,12,13]. Therefore, manufacturers have continually been researching new materials for CAD/CAM utilization, attempting to combine the advantageous properties of ceramics (such as durability and color stability) together with the advantages of composite resins (such as their improved flexural properties and low wear resistance) [14,15].

Popular composite resin blocks include Vita Enamic (VE; Vita Zahnfabrik, Bad Säckingen, Germany) and Lava Ultimate (LU; 3M ESPE, Saint Paul, MN, USA), which have found frequent clinical usage. Vita Enamic is a PICN (polymer-infiltrated ceramic-network) dental material, whereas Lava Ultimate is a nanoparticle-and-nanocluster-filled resin. More recently, new block systems have been introduced, including the Amber Mill Hybrid (AH, Hass, Seoul, Korea), also a PICN dental block, and Katana Avencia (KA, Kuraray Noritake Dental, Tokyo, Japan), a filler press, and monomer infiltration resin-based block. Meanwhile, the lithium disilicate glass ceramic block Amber Mill (AM, Hass, Korea) has also been introduced.

However, the availability of research on these materials is limited, and therefore, their material properties need to be suitably evaluated to better understand their attributes and limitations. Although there is no single attribute that can predict the material clinical lifetime and success or failure, parameters such as flexural strength, flexural modulus, and modulus of resilience can provide insights into the dynamic behavior of these materials under simulated occlusal stress [16]. From a biomimetic perspective, these materials are required to exhibit mechanical properties that approximate those of human enamel and dentin [17,18,19,20]. Simultaneously, the wear properties of these materials also need to be evaluated, because wear resistance is important for lifetime and is the key to maintaining stable occlusal contact over time. Further, rough material surfaces can cause dental caries and a severe wear to the antagonist tooth, the severe wear can even lead to the disappearance of stable occlusion [21,22,23,24,25].

Against this backdrop, the objectives of this study were to determine and compare the biaxial flexural strength, modulus, and wear ability of five different dental restorative milling blocks indicated for chairside fabrication.

## 2. Materials and Methods

**Figure 1:**
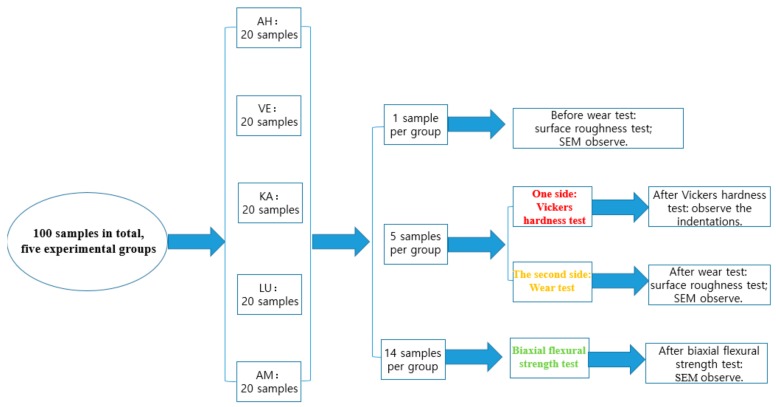
The protocol diagram with the number of samples in each group and sub-group.

Four composite materials and one glass ceramic from four companies were selected for this study. The composition and content of the materials are listed in Table 1. In order to simplify the comparison, A2 or M2 were chosen for all materials, but the color differences between the discs and the manufacturers were still detectable. Based on manufacturer specifications, all the materials had the same filler and/or resin composition or very similar compositions, which provides a basis for the comparison of material groups. The protocol diagram with the number of samples in each group and sub-group are listed in Figure 1.

### 2.1. Specimen Preparation

The blocks of each material were received from the manufacturers, and five groups of samples were prepared in the experiment (20 samples per material). The blocks were first cut into slices and milled to a suitable size of 12 mm in diameter and 1.2 mm in thickness use the milling machine. In order to obtain an optical finish, the samples were first bonded with wax and subsequently polished to remove any machining damage with the use of 1 μm diamond paste (Beuhler Ltd., Lake Bluff, IL, USA), and finally separated by removing wax under high temperature; ultimately, the surfaces of each group were polished to an average level of 0.012 μm. 

To simulate a humid environment similar to the oral environment in which the materials are expected to be present, the most commonly used artificial ageing method, especially in restorative dentistry, is water storage. In order to simulate the hydrolytic degradation of the sample interface components [26,27,28], all samples were cleaned using ultrasonic waves for 1 min and then stored in distilled water at 37 °C for 7 days.

### 2.2. Vickers Hardness Test

The Vickers hardness test was performed according to the C1327 standard using a Mitutoyo microhardness tester (Mitutoyo, Takatsu-ku, Japan) under a force of 1 kg for composite resins and 2 kg for glass ceramics for a constant indenter dwell time of 15 s. We selected two random points on each sample for hardness measurements (five samples per group). All indentation tests were performed under ambient laboratory conditions. After indentation, the hardness value of each material was obtained by calculating the diagonal length of each square indentation. Subsequently, we used an image analyzer (Leica DM 2500M, Leica Microsystems, Wetzlar, Germany) to observe the indentations.

### 2.3. Wear Test

After the hardness test, the second side of the five samples was used for the wear test. According to the ISO/TR 14569-1:2007(E) standard, five reference materials were also to be prepared; the reference material was made from a linear uncrosslinked and unplasticized poly(methylmethacrylate) (PMMA) with a molecular weight over 1,000,000 and the same size as the experimental samples. We first determined the density (ρ) of the five groups of the experiment in accordance with ISO/DIS 18754 and the density (ρ) of reference materials (acrylic resin) in accordance with ISO 1183-1:2019. According to ISO/TR 14569-1:2007(E) standard, the samples were rinsed with distilled water and cleaned for 1 min with the use of ultrasonic waves. Next, they were wiped clean with paper until no moisture was visible. The specimens were then dried in air for 15 s and weighed; this weight was recorded as *m*_1_ (accurate to the order of milligrams).

**Figure 2:**
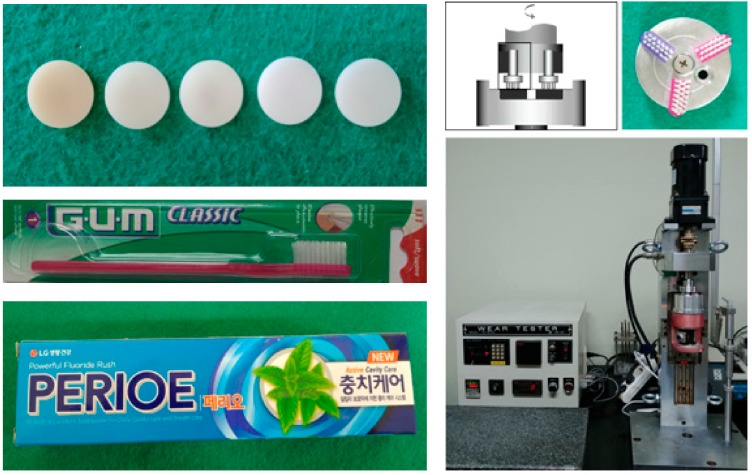
The test machine, toothbrushes, and toothpaste used in the wear test.

The wear performance was measured by application of the toothbrushing test according to the ISO/TR 14569-1:2007(E) standard. The toothbrushing equipment (model K236, Tokyo-Giken Co Ltd., Tokyo, Japan) (Figure 2) consisted of two components: The lower component was a stainless-steel disc with six uniform circular holes, which made it possible to brush five different materials and one reference material in the same group. All six holes had the same diameter as the sample (12 mm) and different thickness than the sample (1 mm); it was ensured that the samples were 0.2 mm higher than the stainless-steel plane to facilitate toothbrush damage when the sample was placed in the disc. The upper section of the toothbrushing machine was used to fix three toothbrush heads angled at 120°, with the long axis being parallel to the samples on the stainless-steel disc. Each toothbrush head was composed of soft nylon bristles (Gum 311, John O Butler, Chicago, IL, USA), and was used under a 150 g load in a direction perpendicular to the sliding surface. Brushing was performed at a frequency of 100–120 rpm/min over a total of 100,000 cycles. Along with toothpaste, distilled water was added every 100 cycles to ensure that the surface remained moist. Toothpaste was mixed with distilled water and Perioe Active Cavity Care Toothpaste (LG Household & Health Care Ltd., Seoul, Korea) in a ratio of 1:2 before the beginning of the experiment. The mixture was shaken evenly before each addition to prevent particles in the toothpaste from being deposited at the bottom. The experiments were carried out at room temperature, i.e., 23 ± 3 °C.

After the toothbrushing test, all samples were ultrasonically cleaned for 1 min, and then weighed as per the same standard; this weight was recorded as *m*_2_ (accurate to the milligram level). In addition, the results were expressed as a percentage reduction in volume.

The brushed surfaces were subsequently observed via scanning electron microscopy (SEM, JSM-6400, JEOL, Tokyo, Japan).

### 2.4. Surface Roughness Test

The surface roughness values of the samples after the toothbrushing test were analyzed using a profilometer (SURFTEST SV-3000, Mitutoyo, Japan). The tester probe was positioned at the center of the sample surface, and the direction for 10 measurements was randomly selected. The measurements indicated the distance between the peak and valley of the sample surface (accurate to 0.01 μm). In order to determine the average roughness (Ra), the measuring speed was maintained constant at 0.15 mm/s and the travelling distance was set to 4 mm with the cut-off length of 0.8 mm. 

### 2.5. Biaxial Flexural Strength Test

**Figure 3:**
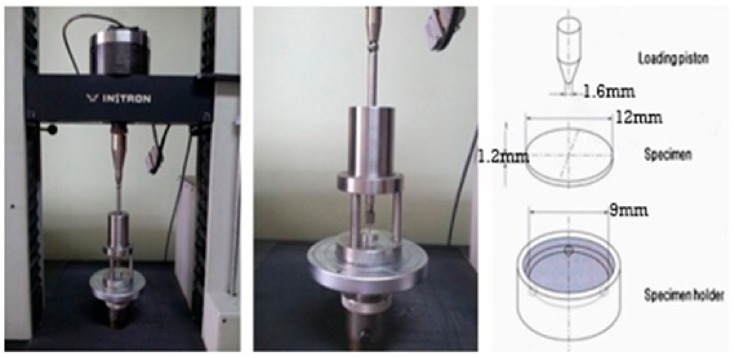
The piston on 3-point biaxial flexural strength test machine and specimen holder size used in this experiment.

We conducted piston-based three-point flexural tests using a universal testing machine (GB 4201, Instron, Wycombe, UK) to measure the biaxial flexural strength of 14 samples of each material according to ISO 6872:2015(E). Each sample was positioned at the center of a metal fixture holder with a diameter of 12.4 mm (Figure 3). Three stainless-steel balls were positioned under the sample to form a circular support with a diameter of 9 mm to ensure uniform transmission of the loading strength to the sample surface. The loading piston tip had a diameter of 1.6 mm, and it was applied at a testing speed of 1 mm/min until the sample broke. The maximum load borne by the sample before breakage was recorded by the test machine and then analyzed as per Weibull analysis. The surface topographies of the broken surfaces were examined by means of scanning electron microscopy (SEM, JSM-6400, JEOL, Japan).

### 2.6. Statistical Analysis

The hardness and surface roughness data were subjected to analysis of variance (ANOVA) followed by the Tukey post hoc test to analyze statistical differences, and the results were considered to be significant when *p* < 0.05. Further, the biaxial flexural strength data were subjected to Weibull analysis to calculate the reliability and lifetime of the materials.

## 3. Results

### 3.1. Vickers Hardness

**Figure 4:**
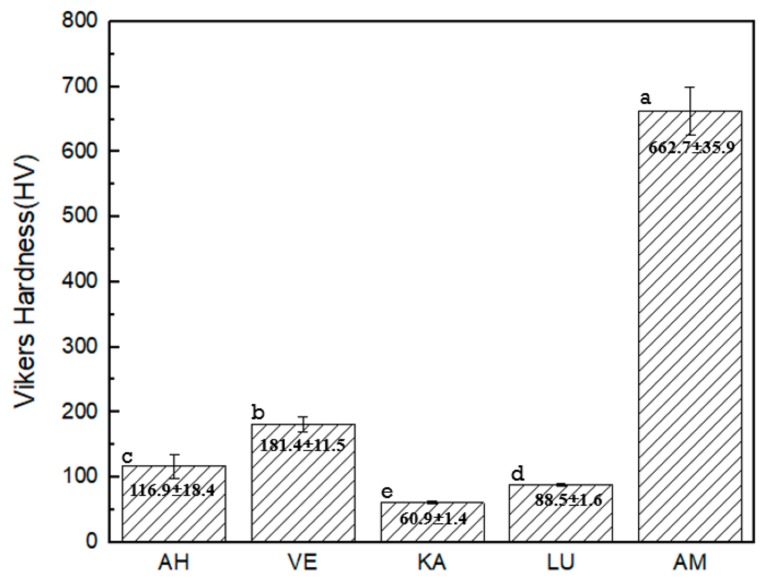
Mean values of Vickers hardness. * Different letters indicate statistically significant differences (*p* < 0.5).

**Figure 5:**
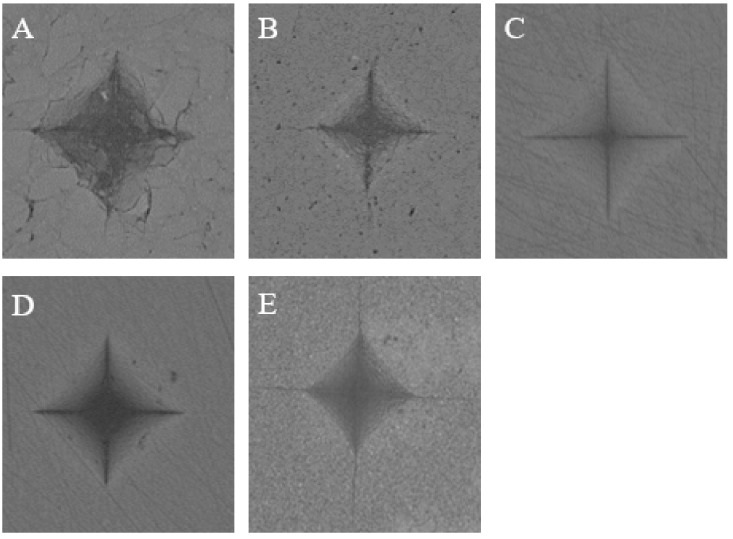
Typical Vickers hardness indentation image of the specimens’ surfaces: (**A**) Amber Mill Hybrid (AH); (**B**) Vita Enamic (VE); (**C**) Katana Avencia (KA); (**D**) Lava Ultimate (LU); (**E**) Amber Mill (AM).

The microhardness values and images of the five materials tested in the study are shown in Figure 4 and Figure 5, respectively. We note that AM exhibits the highest hardness value, while KA exhibits the lowest one. The hardness values range from high to low in the order of AM > VE > AH > LU > KA. A statistical difference is observed between each pair of groups. From the typical Vickers hardness indentation images, we note that only AM, which is a brittle lithium disilicate glass ceramic, shows a crack extension around the indentation, whereas the other four composites exhibit no cracks. However, the AH indentation is not as “neat” as those of the other three composites, which causes fragmentation of small pieces of material around the indentation.

### 3.2. Wear Test

The mean loss in mass, material density, loss in volume, and relative worn volume after the toothbrush test are listed in Table 2. The mass loss and relative worn volume of the tested materials exhibit differences between each other. The two ceramic-based resin materials, AH and VE, exhibit the lowest relative worn volume loss, followed by the lithium disilicate glass ceramic AM. The two-resin based ceramic materials, KA and LU, exhibit higher relative worn volume loss when compared with the other composites.

**Figure 6:**
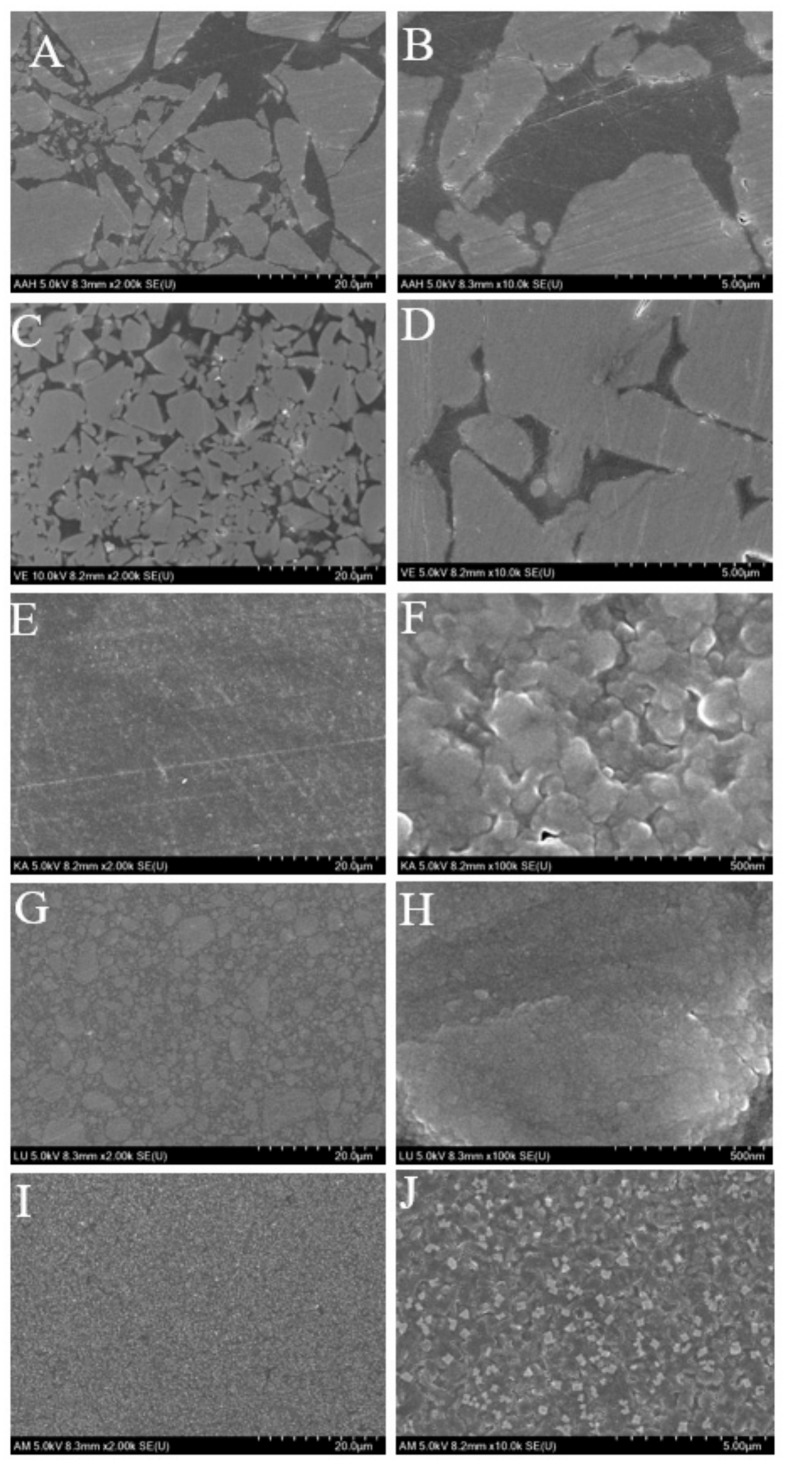
SEM images of the five tested materials before toothbrushing: (**A**,**B**): AH; (**C**,**D**): VE; (**E**,**F**): KA; (**G**,**H**): LU; (**I**,**J**): AM.

**Figure 7:**
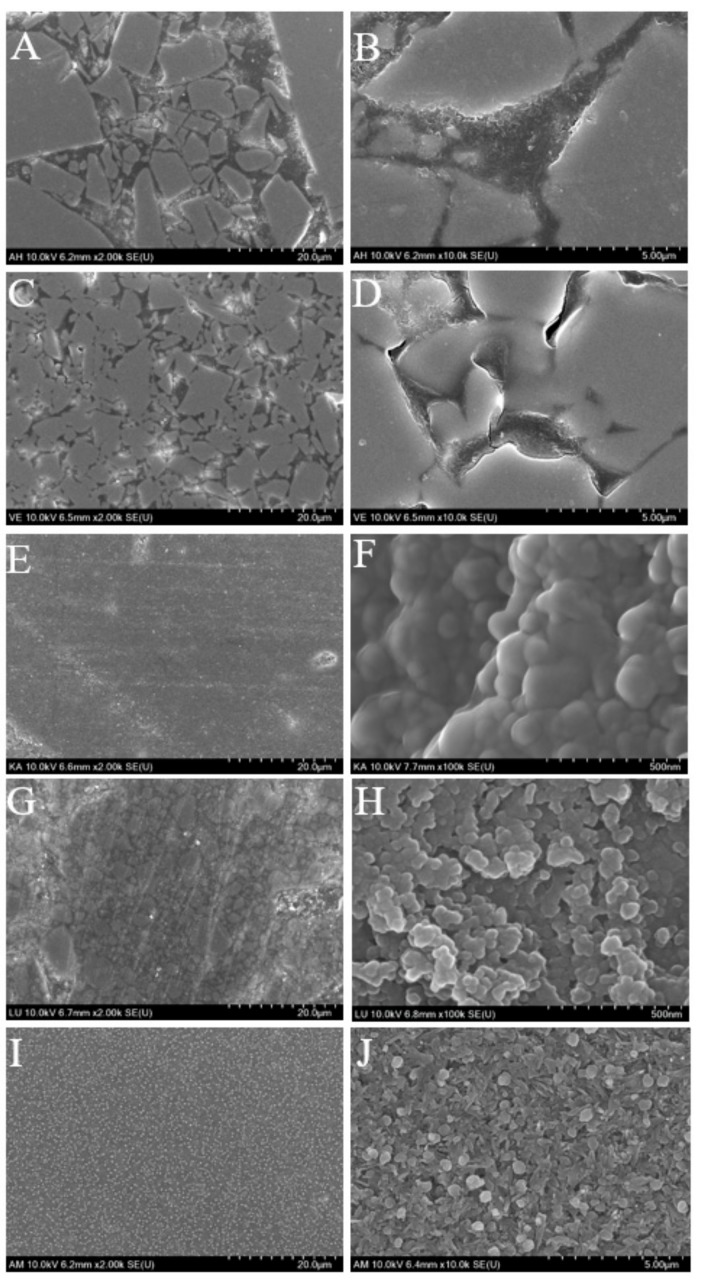
SEM images of the five tested materials after toothbrushing: (**A**,**B**): AH; (**C**,**D**): VE; (**E**,**F**): KA; (**G**,**H**): LU; (**I**,**J**): AM.

Figure 6 and Figure 7 show the representative SEM images of the surfaces before and after toothbrushing. Surface defects of the two ceramic-based resin materials AH and VE can easily be observed. Their surface quality is reduced, the surface appears rough and uneven, and the resin blocks and spherical fillers on the surface appear to be worn away by the toothbrushing, thereby forming deep grooves. The remaining ceramics are also severely worn. However, in contrast, no significant resin loss is observed on the KA and LU surfaces; only a few slight scratches and tiny fatigue cracks are observed. AM exhibits the smoothest surface; the traces of toothbrushing on the surface can barely be noticed.

### 3.3. Surface Roughness after Toothbrushing

**Figure 8:**
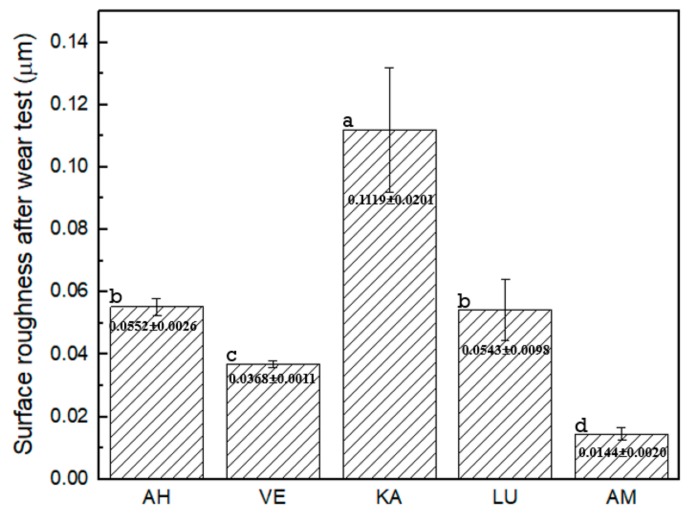
Surface roughness (Ra) after toothbrushing. * Different letter indicates a significantly difference between that two groups (*p* < 0.5).

The mean surface roughness and standard deviations after toothbrushing for the various materials are shown in Figure 8. The surface roughness values (Ra) from higher to lower values can be ordered as KA > AH > LU > VE > AM. When compared with the roughness of 0.012 μm before the toothbrushing test, KA exhibits a significantly higher roughness change than the other materials, whereas AM exhibits almost no change. Except for LU and AH, there is a clear statistical difference between any two other groups.

### 3.4. Biaxial Flexural Strength

**Figure 9:**
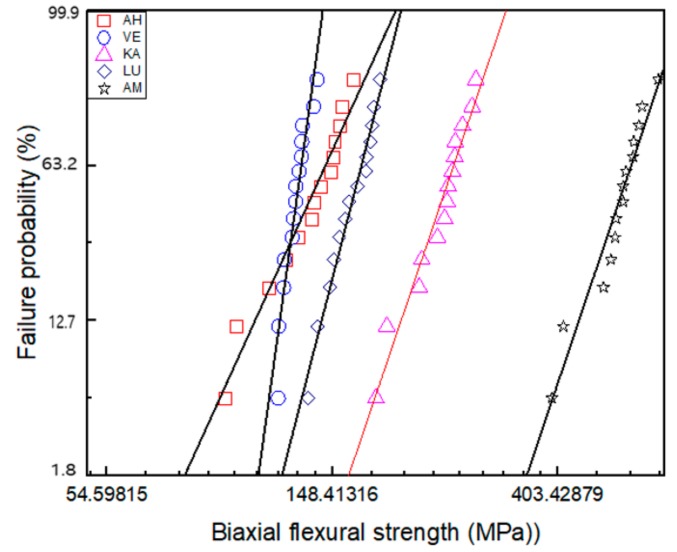
Failure probability according to the Weibull analysis of biaxial flexural strength.

Table 3 and Figure 9 present the biaxial flexural strength information obtained as per the Weibull analysis. Table 3 lists the relevant data including the Weibull modulus, maximum strength, average strength, number of broken pieces, and Weibull distribution regression. We note that VE exhibits the highest Weibull modulus and AH the lowest. As regards the maximum biaxial flexural strength (σ0) and the average biaxial flexural strength (σf), the five materials can be ranked as AM > KA > LU > AH > VE from high to low. Figure 9 shows the calculated data for the fracture results of 14 samples in each group along with the fitting of the data set for each material by means of the least-squares method; the best-fit straight line with the smallest error determines the failure probability line for each material. This is a generalization of the exponential distribution and describes the survival and failure times of these five brittle materials. According to the fitting line, we observe that under the same failure probability, AM can withstand the maximum force, while AH and VE can withstand the least.

**Figure 10:**
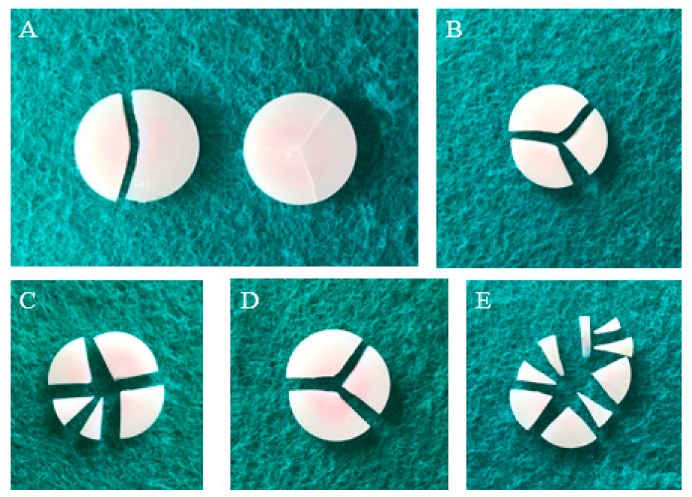
Typical failure mode performed in all groups: (**A**) AH; (**B**) VE; (**C**) KA; (**D**) LU; (**E**) AM.

Figure 10 depicts the typical failure mode for each material. All fractures are fragile failures caused by fatigue. Most of the AM and KA samples appear to be evenly broken into multiple pieces, indicating that they can withstand high strength and high energy, while most of the VE and LU samples are broken into three pieces. Only the AH samples are broken into two pieces, showing a special failure mode. Further, although some samples show failure, the pieces are still connected and not completely separated.

**Figure 11:**
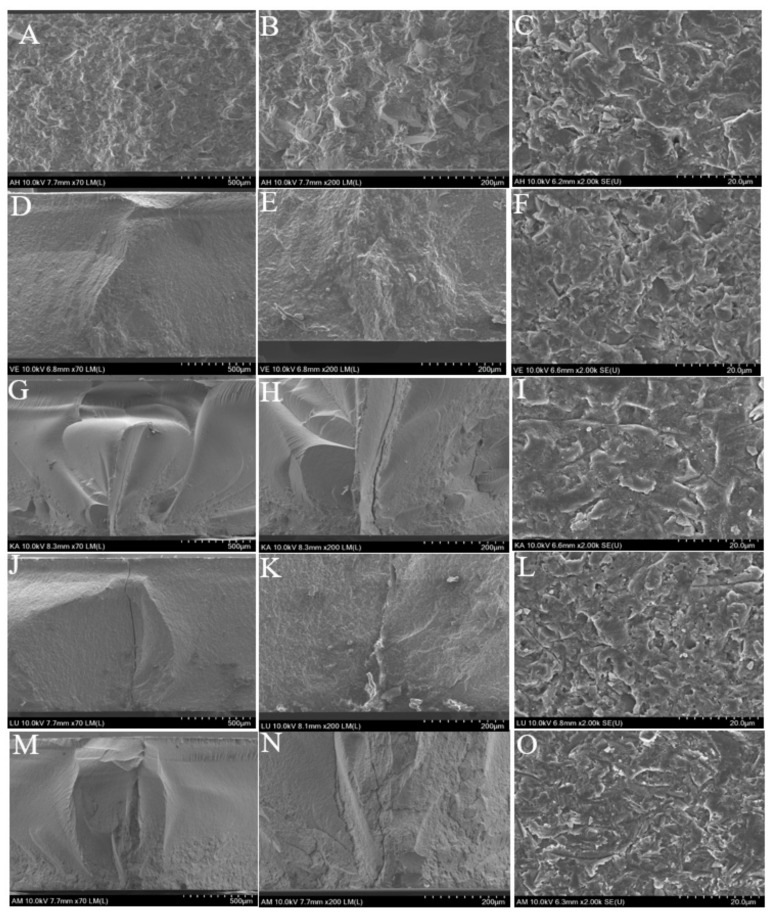
SEM images of fractured surface after biaxial flexural strength test; (**A**–**C**): AH; (**D**–**F**): VE; (**G**–**I**): KA; (**J**–**L**): LU; (**M**–**O**): AM.

Figure 11 shows the typical fracture surfaces and inner layers after the biaxial flexural strength test as observed by SEM. The inner layers of the two ceramic-based resin composites AH and VE do not pass through the full layer of cracks, and the cracks only travel between the joint faces of the ceramic and resin. However, the two resin-based ceramic composites LU and KA show results similar to that of the brittle glass ceramic AM. We can clearly observe that the cracks pass through the full layer of the sample to reach the surface.

## 4. Discussion

In this study, we compared four composite materials and one glass ceramic that are currently used in oral prosthetics. Although laboratory studies cannot fully reproduce the micro-ecological environment in the human mouth, we can simulate an environment that is maximally close to the environment in the mouth to monitor and evaluate the clinical performance of the materials. 

The surface hardness of a material can be considered as the resistance of the material to external indentation. Some studies have suggested that this hardness is closely related to the wear resistance of the material, and it can even form a predictor of wear resistance [29,30,31]. In several other studies, researchers have attempted to confirm the correlation between the hardness and wear resistance of composite resins; however, the results are insufficient to draw any conclusions [29,32]. The hardness value analysis as per two-way ANOVA indicates that the material type and storage conditions are important. Therefore, we performed an immersion test for seven days before the Vickers hardness test was performed. 

The five blocks have different inorganic filler content, as listed in Table 1: The content amount variation can be expressed as VE > LU > AH > KA, while AM is considered as a separate glass ceramic. The results of the Vickers hardness test are similar but not identical to the rank order of the inorganic filler component. The Vickers hardness test results show that although the inorganic filler content of AH is lower than that of LU, its strength is higher than LU. According to manufacturer specifications, LU is a resin-based ceramic nanofill composite, whereas AH is a ceramic-based resin composite. This result indicates that the results of the Vickers hardness test can not only be affected by the content of the inorganic filler, but also the filler size, filling method, and polymerization technique.

As regards the wear resistance, we used toothbrushing to mimic frequent oral abrasions to evaluate the resistance of different materials [33,34]; the samples in this study were brushed 100,000 times, which may approximately equal the amount of in vivo toothbrushing carried out over a period of approximately 4.2 years. Further, we studied the mass loss, volume change, and surface roughness, and we examined the SEM images of the brush-damaged surfaces. Although the 3D surface roughness parameters give a better description of the surface topography, as it is defined in one sampling area while 2D parameters consist of several sampling lengths; the 2D parameter Ra remains a helpful general guideline of surface topography, providing a useful and easy to understand value, which makes comparison with other literature and standards easy. So in this study 2D measurement was still selected. The toothbrushing test affords abrasion between the toothbrush bristles and the toothpaste [35,36], but does not include all of the wear mechanisms experienced by the restorative material in the oral cavity. Other types of wear include food wear, load on the occlusal area of the teeth, and erosion. These factors can lead to the wear of the organic matrix, exposure of inorganic particles, and loss of filler particles. In this study, all materials exhibited a mass loss and volume change after the toothbrushing test. The two ceramic-based resin composites AH and VE exhibited the lowest mass loss and volume change, followed by the glass ceramic AM. The two resin-based ceramic nanofill composites LU and KA exhibited the highest quality loss and volume change. However, although AH and VE showed lower weight loss and volume change, our SEM observations indicated that the two groups of surfaces had large cracks. We attribute this result to the fact that AH and VE are porous cross-linked-ceramic-based resin composites; these materials have a hard ceramic as the substrate, but the resin filler has very low hardness and is therefore easily removed in brushing experiments, thereby leading to the formation of cracks. In contrast, although LU and KA exhibit higher mass loss and volume change, they cannot easily be removed because they are resin-based nanofilled clusters, and therefore, no obvious damage is observed in the SEM images. Here, it is worth noting that the glass ceramic AM exhibits a very small change in surface roughness before and after brush damage; however, it still shows a mass loss of 0.1 mg. We speculate that this result may be related to the dissolution of the AH filler by the fluoride ions of the toothpaste. This line of reasoning also leads to the idea that regarding the improvement of the wear resistance of composite materials, we should focus on both the hardness of the filler and the wear resistance of the resin matrix. In this regard, there are already different manufacturers who have begun optimize material performance; however, more research and investigations are required to assess whether such optimization can promote better dental performance.

Uniaxial strength tests, such as three- or four-point bar specimens have long been used to determine ceramic strengths. Such measurements, however, may provide only a partial characterization of load-bearing capacity. Two advantages are attributed to the piston-on-3-ball biaxial flexural strength test compared with uniaxial strength test; support on three balls ensures contact even with warped specimens. Second, a theoretical stress analysis is available from the general solution. So in this study, biaxial flexural strength test was chosen. Weibull analysis is considered as an acceptable analytical method for assessing the reliability or lifetime of engineering materials. A brittle material, particularly dental material, needs to withstand chewing pressure, and therefore, the material biaxial flexural strength forms an important reference factor. However, due to the uneven distribution of forces in the mouth, a single value cannot accurately reflect the strength of the material. Weibull analysis assesses the variability and reliability of materials by calculating the failure probability of materials at any pressure, which aids in better understanding the properties of materials [37]. A higher m value corresponds to a superior performance of the material; it means that the material is more reliable than one with a lower m value [38,39]. In this study, AH exhibited the lowest m value while VE showed the highest. From Figure 9, we find that although AH has higher average and maximum strength than VE, its fitting-line span is large, which makes it highly unreliable. The biaxial flexural strength of the materials can be ordered as AM > KA > LU > AH > VE. AM, as a glass ceramic that can withstand a force of up to 530 MPa, can be used in the posterior region of the mouth to withstand the chewing pressure; however, the other four composite materials exhibit values far lower than the unilateral chewing pressure of the posterior region and cannot be used in the posterior region [40]; however, these values are still higher than the maximum occlusal force of the anterior teeth [41]. Consistent with the typical failure mode observed in Figure 10, AM and KA, which are subjected to higher strength, are evenly fractured into multiple pieces, while the other three materials are fractured into two or three pieces. In particular, in the AH group, two samples showed different failure modes. The failure results indicated that the sample had already broken, but the pieces were still not separated. We assumed that this was because the brittle ceramic base had broken, but the filled resin was still connected. The SEM images showed that the cracks of the three materials, AM, KA, and LU, travelled straight between the ceramics and spread clearly from the bottom to the surface. On the other hand, the cracks of the two ceramic-based resin composites, AH and VE, spread along the curved interface of ceramic and resin.

Although we observed differences between each material via our testing and SEM observations, we note here that these differences alone cannot be used to perfectly predict the functioning of the materials under clinical conditions. Other factors and the oral environment have to be considered to determine the optimal composite for dental restoration [42,43,44]. In this regard, many clinical studies have reported on the relevant properties of composite materials, but there is still a need for a more comprehensive assessment of the materials before they can be implemented for clinical use.

## 5. Conclusions

From our comparison of five different groups of dental restoration materials, we found that all the tested materials exhibited varying degrees of mass loss and surface roughness. In addition to AM as a glass ceramic exhibits a higher biaxial flexural strength, the other four resin composites are distributed from 138.9 MPa to 281 MPa, therefore these CAD/CAM composite materials are more suitable for single crowns in the anterior region and not recommended for fixed-bridge restorations. Further, the properties of the composite materials are related to the filler content, filler volume, and polymerization methods.

Dental composites are versatile materials that have received widespread attention and application since they were introduced into oral science more than 50 years ago. These materials are constantly being put forward with higher requirements in the process of use, and researchers and manufacturers are constantly developing and researching new products to improve their performance. It is expected that these materials can be further developed to include improved fracture resistance and wear resistance.

## Figures and Tables

**Table 1:** Characteristics of the materials used in the study.

Material	Code	Company	Lot No.	Monomer Composition	Filler Composition	Filler Mass (%)
**Amber Mill Hybrid**	AH	Hass, Kangreung, Korea	Prototype	UDMA, TEGDMA	Crystalline glass	76
**Vita Enamic**	VE	Vita Zahnfabrik, Germany	2M2-T(754101)	UDMA, TEGDMA	Feldspar ceramic enriched with aluminum oxide	86
**Katana Avencia**	KA	Kuraray Noritake, Tokyo, Japan	A2-LT(000015)	UDMA, methacrylate monomer	SiO_2_ (40 nm), Al_2_O_3_ (20 nm)	62
**Lava Ultimate**	LU	3M ESPE, Saint Paul, MN, USA	A2-HT(N826384)	Bis-GMA, UDMA, Bis-EMA, TEGDMA	SiO_2_ (20 nm), ZrO_2_(4–11 nm), ZrO_2_/SiO_2_ clusters	80
**Amber Mill**	AM	Hass, Kangreung, Korea	A2(EBE06KJ0202)	-	Crystalline Lithium disilicate ceramic	-

**Table 2:** Mean loss in mass, material density, loss in volume, and relative worn volume after toothbrushing.

Material	Δm (mg)	ρ (g/cm^3^)	ΔV (mm^3^)	ΔVrelw (%)
**AH**	0.067 ± 0.047	1.927 ± 0.011	0.035 ± 0.025	0.92 ± 0.673
**VE**	0.1 ± 0.082	1.947 ± 0.006	0.051 ± 0.042	1.29 ± 0.955
**KA**	0.267 ± 0.047	2.769 ± 0.032	0.096 ± 0.018	2.977 ± 0.375
**LU**	0.133 ± 0.047	2.107 ± 0.007	0.063 ± 0.022	2.023 ± 0.073
**AM**	0.1 ± 0.001	1.708 ± 0.003	0.059 ± 0.0005	1.893 ± 0.514
**Acrylic (Reference material)**	3.8 ± 1.023	1.141 ± 0.002	3.332 ± 0.901	-

**Table 3:** Weibull analysis of biaxial flexural strength.

	AH	VE	KA	LU	AM
**m**	6.40	20.94	8.58	11.33	8.63
**σ_max_**	163	138.9	281	183.7	631.1
**σ_f_ ± SD**	134.34 ± 6.40	126.34 ± 6.63	240.88 ± 29.62	161.93 ± 11.33	529.46 ± 63.67
**BP**	2.14 ± 0.35	2.93 ± 0.26	5.64 ± 1.23	3.29 ± 0.59	6.36 ± 1.17
**N**	14	14	14	14	14
**R^2^**	0.96	0.92	0.96	0.98	0.94

**m**: Weibull modulus, **σmax**: maximum biaxial flexural strength (MPa), **σf**: average biaxial flexural strength (MPa), **BP**: number of broken pieces, **N**: number of samples, **R^2^**: Weibull distribution regression.
